# Racial/Ethnic Disparities and Immunotherapeutic Advances in the Treatment of Hepatocellular Carcinoma

**DOI:** 10.3390/cancers16132446

**Published:** 2024-07-03

**Authors:** Alexsis Garcia, Stephen O. Mathew

**Affiliations:** Department of Microbiology, Immunology & Genetics, UNT Health Science Center, Fort Worth, TX 76107, USA; alexsisgarcia@my.unthsc.edu

**Keywords:** hepatocellular carcinoma, cancer, immunotherapy, race/ethnicity

## Abstract

**Simple Summary:**

Hepatocellular carcinoma (HCC) is the most common form of liver cancer in adults. For patients with early HCC, surgery, local destructive therapies, chemotherapy and liver transplantation provide curative potential. However, these strategies do not provide significant extension in lifespan often developing resistance to therapy and disease recurrence. Differences in incidence, treatment outcomes, genetic differences, ethnic and socioeconomic background all contribute to the racial/ethnic disparities in HCC. Recently, immunotherapy has revolutionized the treatment paradigm for HCC. Despite these advances it is vitally important to continue research efforts to understand resistance and cancer escape mechanisms in racially distinct populations that could potentially lead to less recurrence, less mortality, and increased overall survival in HCC patients.

**Abstract:**

Hepatocellular carcinoma (HCC) remains one of the leading causes of death among many associated liver diseases. Various conventional strategies have been utilized for treatment, ranging from invasive surgeries and liver transplants to radiation therapy, but fail due to advanced disease progression, late screening/staging, and the various etiologies of HCC. This is especially evident within racially distinct populations, where incidence rates are higher and treatment outcomes are worse for racial/ethnic minorities than their Caucasian counterparts. However, with the rapid development of genetic engineering and molecular and synthetic biology, many novel strategies have presented promising results and have provided potential treatment options. In this review, we summarize past treatments, how they have shaped current treatments, and potential treatment strategies for HCC that may prove more effective in the future.

## 1. Introduction

Hepatocellular carcinoma (HCC) has continued to remain a major health problem globally. In many countries including Southeast Asia, where the incidence of hepatitis B (HBV) and hepatitis C (HCV) is high, HCC is the most common cause of cancer-related death. It is estimated that the incidence of occurrence of HCC will increase in Asia (7.5%), Europe (2.6%), Africa (10%), North America (6.3%), and Latin America/Caribbean (5.3%) by 2040 [1]. Many factors contribute to the development of HCC, which include hepatitis B (HBV), hepatitis C, diabetes, nonalcoholic fatty liver disease (NAFLD), alcohol-related cirrhosis, smoking, obesity, and various other factors [2]. Additionally, comorbidities, epigenetic factors, the tumor microenvironment (TME), and other predispositions also play a role in the development of HCC [3]. 

HCC and many other cancers evolve and can establish various suppressive inhibitory mechanisms that allow host immune evasion and tumor growth/progression [4]. In many instances, cancer cells can reduce the expression of antigen-presenting molecules and neoantigens, MHCI and non-self-antigens respectively, which allow cells to escape immune cell recognition. Additionally, through the release of various molecules (i.e., cytokines, growth factors, and vascular endothelial growth factors (VEGF)), cancer cells can mediate immunosuppression and promote tumor growth/metastasis. The screening and staging of HCC is an important factor in establishing a proper treatment regimen. This process is performed via the Barcelona Clinic Liver Cancer staging system that is partnered with the Child–Pugh score to establish an appropriate baseline for treatment. However, many patients, especially those within ethnic minority groups, do not obtain proper screening and are often missed due to the presentation of symptoms at an advanced stage that is often incurable and inoperable [5].

Currently used treatments include liver transplantation, surgical resection, chemotherapy, radiation therapy, and the use of the multi-kinase inhibitor Sorafenib for advanced HCC patients has improved the standard of care for the treatment of HCC but falls short. Surgical resection is incredibly effective for early-stage HCC; however, even after successful resection, recurrence of HCC often limits survival [6]. This often leads to many patients needing liver transplantation. However, for patients with alcohol-related liver disease, the Milan criteria for liver transplantation exclude them due to poor liver function and reduced overall survival (OS), and the disease is often more advanced when the presentation of symptoms occurs compared to their non-alcohol-related counterparts [7]. Additionally, for patients with advanced cirrhosis, further loss of liver function is intolerable and may result in damage to the liver parenchyma because of resection. Many HCC patients are diagnosed in advanced stages and are often ineligible for such treatment. In some cases, downstaging or decreasing the size of the tumor using locoregional therapies (e.g., transcatheter arterial chemoembolization [TACE]) has been considered for patients to be treated within the Milan criteria after which liver transplantation would be utilized. 

In addition to resections and liver transplants, various chemotherapies have been developed and utilized for advanced HCC patients [8]. The use of Sorafenib, a multi-kinase inhibitor approved by the US Food and Drug Administration (FDA) in 2007, has resulted in increased OS in advanced unresectable HCC patients. Overall survival (OS) for advanced HCC patients when treated with Sorafenib increased from 7.9 months to 10.7 months [8]. Another multi-kinase inhibitor, Lenvatinib, was approved by the FDA which, when compared to Sorafenib, exhibited tolerable rates of adverse effects and was considered to not exacerbate tumor progression [9], with adverse effects being hypertension, diarrhea, decreased appetite, and weight loss [10]. However, despite clinical success, both Sorafenib and Lenvatinib were only able to barely extend survival time by 2–3 months as monotherapies [3], as such novel HCC therapies have been researched extensively.

Other than the various chemotherapies, radiotherapy has also been utilized in the treatment of HCC. RFA, or radiofrequency ablation, was approved for liver lesion application in 1997 [6]. Radiotherapy applies heat to the cancerous tissues (hyperthermia) to induce the destruction of the tumors. This method relies on the normal hepatic liver tissue still retaining its ability to regenerate. The spared tissue that is not targeted can compensate for the radiation-damaged liver [11]. Minimally invasive therapies such as RFA, while instrumental in the treatment of HCC, must continue to be studied to further expand the effectiveness of treatment to the patient population that is eligible to undergo local ablation.

**Figure 1:**
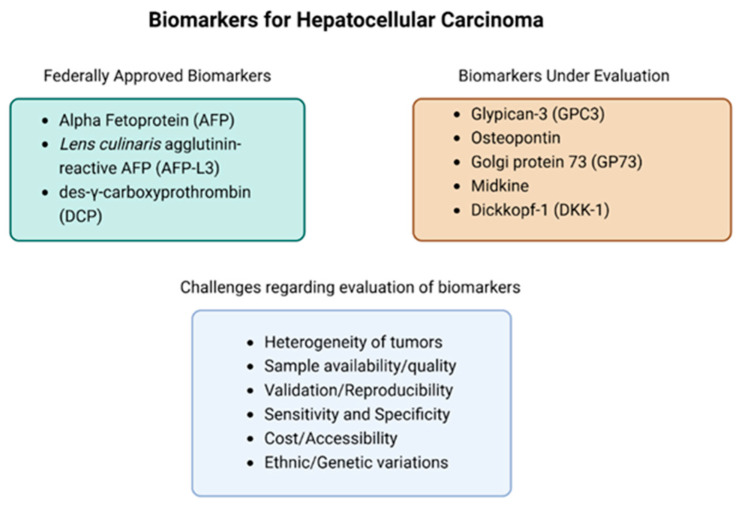
Currently approved FDA biomarkers, biomarkers currently being evaluated in clinical trials, and a list of the challenges associated with evaluating biomarkers.

Some studies have evaluated novel serum biomarkers, while others have been FDA approved for use as potential drug targets. Some of the FDA-approved biomarkers are alpha-fetoprotein (AFP), *Lens culinaris* agglutinin-reactive glycoform of AFP (AFP-L3), and des-γ-carboxyprothrombin (DCP) [Figure 1]. However, these biomarkers have been noted to cause a notable increase in false positives despite their increased sensitivity [12]. As such, many recent studies have begun looking into additional biomarkers such as glypican-3 (GPC3), Osteopontin, Golgi protein 73 (GP73), Midkine, and Dickkopf-1 (DKK-1) [13]. Exonuclease-1 has also shown potential as a prognostic and diagnostic biomarker; however, clinical studies evaluating its efficacy are still needed [14]. In addition, there are non-invasive biomarkers (i.e., microRNA [miRNA]) within the saliva that have been detected in HCC patients [15]. However, many of these novel biomarkers are still being evaluated to ensure their efficacy. The difficulties with developing sensitive, specific diagnostic cancer biomarkers stem from the molecular heterogeneity of individuals and the heterogeneity of cancers. Establishing a baseline “normal” value of any biomarker as well as the lack of a unique marker present in all cancers has presented a fundamental issue in the development of additional biomarkers (Figure 1) [16]. Additionally, certain serum markers like extracellular miRNAs may require more sophisticated and expensive instruments to obtain precise measurements [16]. Similarly, blood-based genomic biomarkers require large-scale validation with large sample sizes to achieve promising results [15]. Many of these challenges require further studies prior to clinical utilization.

## 2. Health Disparities in HCC

### 2.1. Disparities in the Incidence of HCC 

It is known that Asian American, African American, Hispanic, and Native American/Pacific Islanders have a higher incidence of HCC than their Caucasian counterparts [10]. Miller et al. also found that the probability of developing cancer in US Hispanic men and women was 37% and 36%, respectively, while this probability varies across cancer types [17]. Men have significantly increased rates of HCC compared to women with a ratio of 3:1 to 4:1. Although the accurate cause of this disparity is yet to be identified, possible factors include behavior, underlying disease etiology, immune responses, epigenetics, and sex hormone interactions with viral replication [18]. In another study, Li et al. found that when comparing non-Hispanic White (NHW) patients to non-Hispanic Black (NHB) patients, they were most likely to live in countries where the household income was less than USD 65,000 while their non-Hispanic Asian counterparts were less likely to live in those countries [19]. Those of lower socioeconomic status (SES) [18] also had similar results compared to those where healthcare remains difficult to navigate [20] and relies on the patients to coordinate their own care. This socioeconomic/healthcare connection has also been noted by Kim et al., who stated that racial minority patients were more likely to be uninsured or receive public insurance when compared to their White counterparts [21]. This can hinder many patients from receiving care due to language and cultural barriers. As such the connection between health disparities, culture, and healthcare access must be further studied to provide patients with better access to treatment.

### 2.2. Disparities in Treatment Outcomes 

Despite the multitude of treatments, there are various dynamics between epigenetic factors, age, gender, race, and socioeconomic status that affect HCC patient outcomes and contribute to health disparities. In a study by Barzi et al., most HCC cases (61%) received some form of treatment. Of those who received treatment, 72% were White, 68% were Japanese American, 51% were Latino, and 48% were African American [22]. They also showed that the most common underlying etiology was hepatitis C virus (33%), non-alcoholic fatty liver disease (31%), cryptogenic (18%), alcoholic liver disease (12%), and hepatitis B virus (5%). Latinos were also found to have hepatitis C (HCV) as the most common etiology (40.6%) along with African Americans (59.5%). Based on this study, it was found that when compared to their White counterparts, African Americans with HCC had higher mortality [22]. Other studies have shown HCC to disproportionately affect individuals of low socioeconomic status (SES), with lower observed surveillance and survival rates in those with the lowest income [18,23,24]. This suggests that treatment, ethnic background, genetic differences, and socioeconomic background all contribute to the disparities in HCC. 

### 2.3. Underrepresentation of Racial/Ethnic Minorities in Clinical Research 

Recently, Monge et al. reported that Hispanics and non-Hispanic Blacks when compared to their White counterparts were severely underrepresented in phase III clinical trials for advanced liver cancer. They used thirteen studies that reported on race and ethnicity and found that 2% (*n* = 184) were Hispanic, 1.8 (*n* = 158) were non-Hispanic Black individuals (NHB), 41.6% (*n* = 3691) were non-Hispanic White individuals (NHW), and 49.8% (*n* = 4413) were Asian individuals. When compared to incidence rates, enrollment of Hispanic individuals who presented with HCC in clinical trials remained increasingly low and static [25] whereas NHW and Asian individuals were overrepresented in liver cancer trials compared to their incidence rates. This underrepresentation in clinical trials may contribute to health disparities within racially distinct populations. However, when assessing immunotherapy efficacy in a study focused on Bronx residents, Hispanics were found to survive longer than non-Hispanics after treatment with ICI [26]. Underrepresentation of racial/ethnic populations in clinical trials is a major contributing factor to health disparities that impede testing of the safety and efficacy of new therapeutic agents in liver cancer.

### 2.4. Biomarkers 

While it is understood that such health disparities contribute to incidence and cancer-related deaths, further studies investigating differential biomarkers along with somatic mutations partnered with the tumor microenvironment within racially distinct populations are needed to understand the various contributing factors of HCC progression. Some biomarkers have been noted to have some variation across racially distinct populations. One study by Khamis et al. found that *CYP2D6* gene expression in Asian HCC patients was higher than that of their Caucasian counterparts [27]. The study profiled 19 common differentially expressed genes between Asian American (AS) and Caucasian American (CA) HCC patients. In addition, IPA (Ingenuity Pathway Analysis) network analysis showed an association between CYP2D6 and HNF4A, a highly expressed receptor in the liver known to regulate development and metabolic liver function. As such, further assessments of CYP2D6 in liver cancer patients are still needed, especially within racially distinct cohorts [27]. In other cases, somatic mutations may contribute to health disparities according to some studies. Some somatic mutations, specifically within cell-free DNA (cfDNA), in Hispanic HCC patients have been found to drive disease progression. In a study by Jiao et al., targeted sequencing of over 262 cancer-associated genes collected from 27 Hispanic patients with HCC revealed that disease progression correlates with the increase in the concentration of cfDNA and mutations. Additionally, they also found that the number of mutations and concentration of cfDNA directly correlated with patient outcomes [28]. Moreover, they also noted that previous studies on cfDNA found that it holds potential early diagnostic value through the use of an assay combining various mutations, along with serum marker AFP, DCP, and HBV integration sites [29]. The study found that this assay had a sensitivity value of 85% and a specificity value of 93%. cfDNA may present as a potential biomarker; however, additional studies must be conducted to evaluate its efficacy.

### 2.5. Risk Factors for Racial/Ethnic Disparities in HCC

Although the majority of HCC (over 90%) occurs with chronic liver disease with cirrhosis of various etiologies, more recent data demonstrate a shift of underlying etiologies of HCC mainly due to the high prevalence of metabolic conditions that include obesity and diabetes [30]. National Health and Nutrition Examination Survey (NHANES) data from 2017–2018 show the prevalence of overweight/obesity to be highest in Non-Hispanic Black individuals (49.6%), followed by Hispanic (44.8%), Non-Hispanic White (42.2%), and Asian (17.4%) individuals [31]. Some studies have found that age and ethnicity play a role in the metastasis of lung and bone cancers, with younger age being associated with a higher risk of metastasis; however, the opposite was seen with Caucasians where increasing age was associated with lower rates of multiple metastatic sites [32]. While these studies assist in understanding certain etiologies of cancer, further studies on HCC patients are required to understand the various risk factors that can contribute to disease progression/development. 

## 3. Treatments for Hepatocellular Carcinoma

### 3.1. Radiofrequency Ablation 

Radiofrequency ablation (RFA) has been an exceptional tool in the treatment of early HCC. While there are additional locoregional therapies such as percutaneous ethanol injection (PEI), percutaneous acetic acid injection (PAI), and cryoablation, radiofrequency has increasingly been utilized for the treatment of early to advanced-stage HCC. Clinicians have seen favorable outcomes utilizing RFA with other ablation treatments in patients with resectable HCC [6]. In addition, radiotherapy has undergone a wide variety of technological advances ranging from whole-liver widefield low-dose irradiation to hyper-fractionation radiotherapy [11]. As these advancements have increased, the risk of complications has been significantly reduced. More recently used radiation therapies include three-dimensional conformal radiotherapy (3DCRT), intensity-modulated radiation therapy (IMRT), volume-modulated arc therapy (VMAT), and stereotactic body radiotherapy (SBRT). Many of these therapies have assisted in the increased use of radiotherapy in the treatment of both early and advanced HCC, some in combination with other therapies such as immune checkpoint inhibitor (ICI) treatment. With 3DCRT, the use of radiotherapy treatment planning software to calculate dose in patient models based on CT scans allows for the careful planning of dosage. It was found that this particular method had the best outcomes in patients with single, small HCC tumors compared to their counterparts with larger multifocal cancers [33]. IMRT was developed based on 3DCRT and uses optimization software based on multi-field irradiation and allows the dose intensity to be varied and more evenly distributed in the target area which helps in the protection of surrounding organs and normal tissues. However, this radiotherapy requires longer exposure time and can have poor tolerance in severe patients. In addition, it was found that when IMRT is conducted following a hepatectomy, survival outcomes were improved compared to other treatments [34]. 

VMAT, when compared to IMRT and 3DCRT, is superior when it comes to dose conformity and has the advantage of delivering irradiation via a rotating gantry while providing lower doses and a reduction in radiation-induced liver injury [35]. SBRT combines the technology of stereotactic therapy and 3DCRT to accurately target the tumor area with a high dosage while decreasing the dosage outside the target area. This technique is most successful in early tumors and small cancers. In several studies evaluating the effectiveness of SBRT, one study found that with non-surgical and non-metastatic HCC patients, the SBRT-treated group showed a 97.4% progression-free survival (PFS) rate within 1 year and an 83.8% PFS rate within 2 years [36]. While many of these radiotherapies have demonstrated increased OS with HCC patients, when used in conjunction with additional treatments including dendritic cell injection and ICI therapies, they promote a stronger immune response when compared to ablation or immunotherapy alone [37].

Although many studies have demonstrated positive antitumor response, there are a few studies that have reported that RFA may promote tumor recurrence in HCC. In addition to an aggressive outgrowth of residual hepatic micro-metastasis [37], there has been evidence of a hypoxic microenvironment that enhances invasive, chemo-resistant tumor cells in HCC post RFA [38]. As such, further studies must be conducted to not only fully assess the efficacy of combinational therapies with radiofrequency ablation but also to reduce residual effects on surrounding tumor/cancerous tissues. 

### 3.2. TAE/TACE

**Figure 2:**
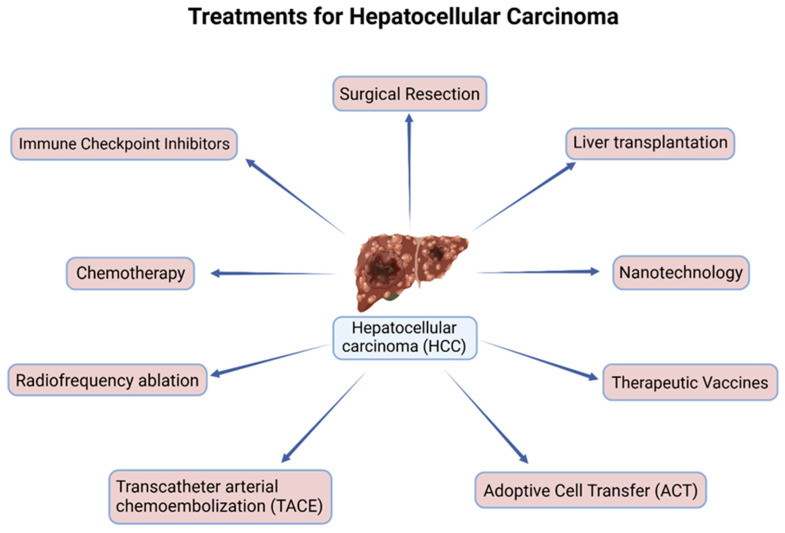
Current treatments for hepatocellular carcinoma (HCC).

Transcatheter arterial chemoembolization (TACE) is one of the most commonly used treatments for HCC for patients with multifocal HCC or tumors unable to undergo resection or ablative therapies [8]. Embolization treatment works to block the blood supply to tissues; however, its use in blocking tumor blood supply has achieved new outcomes in the treatment of unresectable HCC [39]. As more studies began to surface, TAE (transarterial embolization) was adapted and utilized to increase its efficacy through the use of chemotherapeutic drugs. In 1981, the use of Ivalon particles was found to be safe for hepatic artery embolization [40]. Later, a new treatment modality was introduced; TACE using an autologous blood clot to treat unresectable HCC patients. The initial use of an autologous blood clot embolization to treat an arterial bleed within the stomach was a nonsurgical procedure. In the study by Gunji et al., they reported that chemoembolization using an autologous blood clot for unresectable HCC was more effective than conventional chemoembolization with improved overall survival and fewer side effects [41]. Although TACE is the standard treatment for intermediate-stage HCC, which includes multiple tumorous lesions confined to the liver without vascular invasion in a patient with preserved liver function and good performance status, its efficacy is limited due to the extensive heterogeneity within cohorts [42]. As such, improvements like the use of TACE in combination therapies have been explored and utilized (Figure 2).

### 3.3. TACE Combination Therapies

In more recent years, TACE has been used in conjunction with other therapies such as radiofrequency ablation (RFA), radiation therapy (RT), and systemic therapies including anti-angiogenic therapy, immune checkpoint inhibitors, and the various available CAR therapies [43]. Yoon S.M. et al. evaluated the efficacy of combination therapy of TACE with radiation therapy and found that the median survival rate of patients who had stable or responding HCC was 19.4 months [44]. Further studies are still evaluating the efficacy of TACE combination therapies due to the heterogeneous nature of HCC. 

### 3.4. Immune Checkpoint Inhibitors 

Recently, immunotherapies utilizing immune checkpoint inhibitors (ICIs) have been incredibly promising in treating HCC (Figure 2). Many ICIs include antibodies targeting cytotoxic T-lymphocyte-associated protein 4 (CTLA-4), programmed cell death protein 1 (PD-1), and programmed cell death ligands 1 and 2 (PD-L1 and PD-L2, respectively), which target and block the associated proteins that decrease immune activity and allow the immune system to detect and target HCC cells [2]. Sorafenib and Lenvatinib, as previously stated, only extended the life span of HCC patients by a few months [45]. As such various studies have continued to evaluate the efficacy of additional immune checkpoint inhibitors in combination with other therapies due to the increased efficacy of ICI treatments. Some additional combination therapies include tremelimumab plus durvalumab, Sorafenib, and ipilimumab, along with several trials assessing nivolumab and ipilimumab in combination with Sorafenib being underway, as shown in Table 1 [46,47,48].

#### 3.4.1. Anti-VEGF Therapy

Vascular endothelial growth factor (VEGF) overexpression was found to be associated with the recurrence of HCC with higher VEGF expression in HCC samples that overexpressed cancer stem cell markers (CSC) in comparison with those that expressed lower levels of CSC [49]. The effectiveness of a combination of the anti-VEGF monoclonal antibody (mAb) Bevacizumab along with Atezolizumab (anti-PD-L1 mAb) in the IMbrave150 trial was shown to be superior to Sorafenib [50]. This combination is now FDA-approved as a first line of treatment for unresectable HCC in the United States and Europe [51]. Atezolizumab [50] targets PD-L1, which prevents interaction with the receptors PD-1 and B7-1, while bevacizumab targets VEGF, which inhibits angiogenesis and tumor growth. 

#### 3.4.2. Anti PD-1/Anti PD-L1 Therapy

Nivolumab and Pembrolizumab are monoclonal antibodies that target PD-1. Nivolumab, in 2017, was given expedited approval for second-line treatment for patients with severe HCC after failure of Sorafenib therapy [52]. During monotherapy treatment with Nivolumab, patients showed a manageable and acceptable tolerability. In another study comparing Nivolumab and Sorafenib as monotherapies, they found that monotherapy of Nivolumab did not significantly prolong survival; however, it did demonstrate favorable clinical durability with increased long-term survival. As such, in more recent studies, Nivolumab has been studied in combination with other procedures to evaluate its efficacy and safety [48]. A randomized clinical trial to evaluate Nivolumab in combination with Ipilimumab, an anti-CTLA-4 mAb, showed manageable safety and promising durable responses [47]. This led to accelerated FDA approval of Nivolumab in combination with Ipilimumab in the US. 

#### 3.4.3. Anti-CTLA-4 Therapy

Ipilimumab, a CTLA-4 inhibitor, was originally used in malignant melanoma and renal cell carcinoma and received approval by the FDA for use as therapy in patients with severe melanoma in 2011 [53]. However, greater incidences of toxicities like rash and colitis appeared in patients as compared to those treated with other immunotherapy drugs like anti-PD-1/PD-L1 [51]. As previously noted, various combinations of ICI therapies have been studied; however, due to the heightened cytotoxicity of some ICI treatments, further combinational strategies are currently being explored in clinical trials.

#### 3.4.4. Immune Checkpoint Resistance

Despite the improved efficacy of ICI drugs in other cancers, HCC remains incredibly difficult to treat due to the rise in ICI resistance. One factor implicated in ICI resistance is the tumor microenvironment (TME). Tumor cells with altered expression of antigen-presenting molecules and a reduced number of cytotoxic effector cells within the TME can drive the development of resistance. Oweida et al. reported that the upregulation of TIM-3 on CD8+ T cells and Tregs in murine models of head and neck cancer when treated with PD-1 blockade in combination with radiotherapy was associated with the development of resistance [54]. In another study using mouse ovarian cancer models, anti-CTLA-4 or anti-PD-1 mAb treatment was associated with the upregulation of LAG-3 on CD8+ T cells, which suggested a potential resistance mechanism [55]. Additionally, apoptotic Tregs were found to be responsible for inducing such resistance to anti-PD-L1 mAb treatment through the increase in extracellular adenosine levels which would in turn suppress the proliferation and subsequent function of T effector cells [56]. With the various mechanisms of resistance reducing the effectiveness of ICI therapy, many additional therapies have been used in conjunction with ICI treatment. 

### 3.5. Adoptive Cell Transfer (ACT) 

Many studies have covered a vast array of treatments, but a treatment modality that has held great promise in the last few decades is adoptive cell transfer (ACT). Various cells such as T cells, dendritic cells, and natural killer cells have been modified to increase their immune response within HCC [57]. It is evident that within HCC, there are various mechanisms by which cancer cells evade the host immune response. As such this allows tumor cells to proliferate and remain undetected by immune cells. 

#### 3.5.1. CAR-T Cells

Chimeric antigen receptor-T (CAR-T) cell therapy has been a focus in recent years. CAR-T cells can recognize tumor-associated antigens (TAAs) and allow for the targeting of the specified tumor cell [58]. CAR-T cells are T cells that have been genetically modified to express an antigen-specific, non-MHC-restricted receptor. The three main domains of CAR are the extracellular antigen-binding domain, the transmembrane domain, and the cytoplasmic signaling domain. The receptor is composed of a single chain variable fragment (scFv) of an antibody, which is fused to a transmembrane domain and a signaling domain [59]. 

There are ongoing studies targeting GPC-3, MUC-1, and CEA, which are HCC-associated antigens [60]. Alpha-fetoprotein is another HCC-associated antigen that has also been evaluated and targeted by CAR-T cell treatment; however, AFP is also lowly expressed in normal hepatic liver tissues. While many recent studies have evaluated the safety and efficacy of CAR-T cells, there is still the immunosuppressive tumor microenvironment as well as the heterogenicity of HCC that hinder CAR-T therapies [3]. 

CAR-T cell therapy has been highly successful in hematological cancers, but its success in solid tumors has been hampered by the immunosuppressive tumor microenvironment (TME) [61]. Previous studies have noted that combination therapies could prove useful in circumventing the immunosuppressive tumor microenvironment [62]. As such, various recent studies have combined additional therapies such as CAR-T with ICIs. Bispecific GPC3/PD-1 CAR-T cells exerted enhanced tumor-suppressing effects in HCC compared to their single target counterparts, providing a path for future treatment for solid tumors through combination CAR-T therapy [59]. 

#### 3.5.2. NK Cell Therapies

Natural killer cells are innate lymphoid cells that provide a cytolytic function in host defense against virally infected and cancerous cells. NK cell sites of development include the spleen, liver, lymphoid organs, and the uterus [63]. NK cells do not require prior antigen exposure to elicit their effector functions [64]. NK cell function is dependent on the balance of inhibitory and activating signals to generate their effector function. Several inhibitory receptors such as the killer cell immunoglobulin-like receptor (KIR) and leukocyte immunoglobulin-like receptor (LILR) families in humans, the Ly49 family in mice, and the CD94/NKG2 receptor family found both in mice and humans and non-MHC-I-recognizing receptors such as killer cell lectin-like receptor G1 (KLRG1), T cell immunoglobulin and ITIM domain (TIGIT); sialic-acid-binding immunoglobulin-like lectin (Siglec), carcinoembryonic Ag cell adhesion molecule 1 (CEACAM1), NKR-P1(A/B), and the Tyro3, Axl, and MerTK (TAM) receptors play a role in tumor immune surveillance. On the contrary, activating receptors like CD16, NKG2D, natural cytotoxicity receptors NKp46, NKp30 and NKp44, 2B4, DNAM-1, NTB-A, and CS1 play a significant role in tumor inhibition [65,66,67,68,69,70]. There are various sources of NK cells like autologous or allogenic NK cells, stem cell-derived NK cells, and NK cell lines for adoptive therapies. NK cells, particularly allogeneic NK cells, can interact with T cells during adoptive transfer, which can lead to graft-versus-host disease (GVHD); as such, the removal of T cells is required prior to therapy [71]. NK cell-mediated treatments have displayed incredible results in tumor immunotherapy; however, tumor cells can also develop several mechanisms to escape NK cell recognition during tumor progression [66]. 

Due to this, various studies have investigated several strategies to modify NK cells to improve their efficacy. One of which involves the expansion of NK cells through the use of IL-2 in combination with other cytokines including IL-21, IL-12, and IL-18. A two-phase expansion protocol that used in vitro NK cell stimulation with IL-15 with a short exposure to IL-21 displayed promising results against Rhabdomyosarcoma [72]. In addition to the combination of cytokines to induce NK cell-mediated immune response, there have been clinical studies evaluating CAR-NK cells after the successes of CAR T cell therapies. In Saetersmoen et al.’s study, CAR-NK cells were found to have potent effector functions; moreover, CAR engineering did not affect NK receptor repertoire [73]. Additionally, due to their shorter lifespan, CAR-NK cells can lower the danger of tumor transformation and autoimmune response [51]. Recent studies examining the use of CAR-NK therapy targeting CD44v6, HER2, TF, B7-H6, EGFR, and PD-L1 against different types of breast cancers have shown promising results [74]. A recent study utilized human epidermal growth factor 2 (HER2)-specific CAR-NK cells generated from ex vivo expanded NK cells from breast cancer patients and healthy donors to assess the safety and efficacy of CAR-NK cell treatments. The HER2-targeted CAR-NK cells were found to have stronger antitumor effects in HER2-positive breast cancer compared to their CAR-T cell counterparts [75]. Additionally, similar results were seen using pancreatic cancer-directed CAR-NK cells augmented with specific chemokine receptors [76]. Yoon, J.H. et al. utilized tumor-mimetic organoids to investigate the synergistic effects of C-X-C motif chemokine receptor 2 (CXCR2)-tethered CAR-pNK cells using three different primary natural killer (pNK) populations: SS-pNK, SS-CXCR1-pNK, and SS-CXCR2-pNK. The study found that the SS-CXCR2-NK cells exhibited significantly enhanced organoid-killing efficacy compared to the other pNK cell populations [76]. Increasing evidence of the use of CAR-NK cell therapies in solid tumors warrants further studies to evaluate CAR-NK cell treatments in HCC. 

#### 3.5.3. CAR-NK/T Therapies

Similar to the various CAR therapies, CAR-NKT therapies have great potential in the treatment of HCC. CAR NKT therapies have the features of NK cells as well as T cells. NKT cells are a powerful tumor cell-targeting tool due to their increased cytolytic activity and their infiltration ability into the TME [77]. In addition, these cells can activate other immune cells (T cells and NK cells) via the production of interleukin 2 (IL-2) [78]. 

There are various types of NKT cells; the most famous NKT is invariant NKT (iNKT). This cell type continues to express factors that are dependent on T cells for maturation, CD4 and CD8, which further divides this type into three subtypes: CD8+, CD4+, and double negative (CD4-, CD8-, or DN) cells [79]. CD8+ and DN types can produce Th1 cytokines while CD4+ uniquely produces Th2 cytokines. NKT cells can be activated in different manners. Similar to T cells, they can be activated by the TCR and recognition of CD1d, but they also can be activated through the expression of killer cell immunoglobulin-like receptors (KIRs) as well as cytokines like IL-12 [77]. In comparison, NKT cells express a limited range of TCR chains (alpha and beta) and instead of recognizing CD1d on MHC, they recognize CD1d on APC cells. An interesting feature of NKT cells is their capability to regulate the immune response better than other immune cells due to the extensive expression of CD1d [80]. There are some challenges as well since impaired lipid synthesis hinders the anti-tumor effect of tumor-infiltrating iNKT cells. The efficacy of iNKT cell-based immunotherapy against tumors can be enhanced by restoring lipid synthesis via activating PPARγ, which helps in triggering αGC-induced IFN-γ production [81]. 

A study by Simon et al. found that the use of chondroitin sulfate proteoglycan 4 (CSPG4)-specific CAR-NKT cells produced significant amounts of pro-inflammatory cytokines capable of specifically lysing human melanoma cells [82]. Another study found that metastatic neuroblastoma (NB) was susceptible to CAR-GD2 NKT cells. Using a NB xenograft model to evaluate CAR-GD2 NKT cells, the results demonstrated that while having incredibly potent antitumor activity, unlike their CAR-GD2-T counterparts, the NKT cells did not induce GVHD [83]. Despite these results, studies are greatly limited by insufficient infiltration into the TME and the low presence of NKT cells in human blood, which poses a challenge in generating consistent and substantial quantities of allogenic NKT cells suitable for CAR engineering [84]. The expansion of NKT cells to circumvent the limited quantities is currently being studied but still requires efficient processing and manufacturing [77].

#### 3.5.4. Dendritic Cell Therapies

Along with the other adoptive cell therapies, the use of dendritic cells has also been evaluated in many studies. Dendritic cells (DCs) are antigen-presenting cells that can activate T cells. After DCs pick up antigens, they migrate to a regional lymph node where they become mature and then deliver the antigen to naïve T cells [85]. While DCs play a critical role in the immune response, they also play a critical role in carcinogenesis/tumor development. This role was noted by the primary mechanisms, tumor antigen tolerance, and T cell inhibition, with the expression of checkpoint ligands or through the release of mediators [86]. In 2010, an important subtype of DCs was identified; these cells have high expression of CD141 and Toll-like receptor 3 [87]. Adoptive cell therapies utilize genetically modified T cells, along with tumor-infiltrating lymphocytes and cytokine-induced killer cells (CIKs). Like other immunotherapies utilizing immune cell responses, more studies are currently examining the combinations of various treatments to evaluate the efficacy of such treatments and circumvent the TME. One meta-analysis found that the combination of DCs and CIKs along with TACE demonstrated prolonged survival time as well as decreased tumor recurrence [88]. Additionally, DCs have also been implemented in therapeutic vaccines to increase tumor infiltration, which will be discussed later in this review. 

### 3.6. Therapeutic Vaccines

Many of the current vaccines that are used for HCC consist of peptides, DCs, and oncolytic viruses. Therapeutic vaccines, especially when used for cancers caused by viruses like hepatitis B and C, can induce antitumor immune responses. For example, the oncolytic vaccine Pexa-Vec when used in high doses displayed improved survival of HCC patients to 14.1 months in comparison to 6.7 months for those on low-dose treatments [89]. Cancer peptide vaccines stimulate the adaptive immune system via TAAs to induce cytotoxic T cell activation and proliferation and kill cancer cells. Peptides, small protein fragments, can often serve as antigens for immune system recognition. The purpose of utilizing such vaccines is to reduce the risk of off-target effects through the exploitation of the high specificity of peptide vaccines. Many studies have delved into exploring peptide vaccines to target HCC-associated antigens. One of which is glypican-3 (GPC3), which was explored in a phase I clinical trial [90]. Thirty-three patients underwent GPC-3 vaccination to assess the safety and efficacy of GPC3 peptide vaccination. None of the patients that participated in the study dropped out from adverse effects; however, it was noted that one patient could not undergo CT scans due to tumor progression after the third vaccination and two other patients discontinued the regimen after the second vaccination due to liver function impairment. Despite this, evidence suggests that the GPC-3 peptide vaccine was well-tolerated with fewer adverse effects. Moreover, while grade III hematologic adverse effects were observed in 4 patients, the effect and safety evaluation committee judged that those events were not related to the administration and subsequent treatment with the peptide vaccine.

Dendritic cell (DC) vaccine formulations involve the isolation of autologous peripheral blood mononuclear cells in vitro, and DC expansion via co-stimulating factors before incubating the mature DCs with specific TAAs or autologous tumor lysate. After the generation of the primed APCs, they can then be infused back into the host for stimulation of an adaptive cell-mediated response. Early clinical trials have shown DC vaccines to be safe and well tolerated when targeting TAAs on HCC cancer cells. In addition, they have also been shown to have improved survival outcomes [91]. The evidence from various pre-clinical studies and clinical trials on DC-based vaccines indicates that they could be an effective therapeutic approach to treat patients with HCC whether given as a monotherapy or as in combination with other anti-cancer therapies [92]. As such, DC vaccines have also been evaluated in combination therapies. In a phase I trial conducted by Wang X et al., HCC patients underwent standard hepatic resections and post-recovery leukapheresis to obtain PBMC where DCs were derived and then later underwent TACE therapy approximately 1 week after leukapheresis. Vaccination with autologous DCs and irradiated tumor stem cell (TC) lines (DC-TC) was not associated with the worsening of Hep B infection nor was it associated with hepatic inflammation or liver dysfunction exacerbation, providing evidence for the need for further studies [93]. 

### 3.7. Nanotechnology

Recently, nanotechnology has been increasingly utilized to improve the activity of drugs by optimizing the drug’s size as well as surface properties to effectively deliver the drug to the site of the tumor. In addition, various approaches have been shown to alter current combination therapies and improve permeability, retention, and pharmacokinetic profiles, which in turn reduce the amount of side effects. Nanotechnology combines the most effective medication with an altered drug administration/delivery method to improve outcomes for various malignancies. Nanotheranostic modalities have the potential to provide a more personalized and targeted approach to cancer therapy. Some recent studies have reported that nano delivery utilizing intravenous drug administration was more effective in comparison to oral administration for tumor inhibition [94]. In addition, Yang et al. evaluated the use of engineered bacteria in conjunction with nanotechnology, specifically the use of ASEc@PNPs. The ASEc@PNPs resulted in lower cancer cell viability compared to PNPs after 24 h [94]. This suggested that the ASEc@PNPs have increased antitumor activity, evident from the co-incubation of HepG2 cells with the ASEc@PNPs compared to PNPs alone.

In a study performed by Wang et al., iRGD-decorated polymeric nanoparticles of Vandetanib were evaluated for delivery in vitro and in vivo for HCC patients [95]. Vandetanib (Vanib) is an FDA-approved oral angiogenesis inhibitor that is capable of inhibiting several tyrosine kinase inhibitors, such as endothelial growth factor (EGFR) and vascular endothelial growth factor (VEGF), that is used to manage medullary cancer [96]. However, like many other oral treatments, low bioavailability influences its therapeutic effects and patients’ overall survival (OS). As such, Wang et al. sought to utilize nanoparticulate delivery mechanisms to increase the efficacy of Vanib and enhance solubility. They hypothesized that by using Vanib and its hydrophobicity as a hydrophobic core to encapsulate Vanib in a biodegradable amphiphilic block copolymer (e.g., poly(ethylene glycol)-block-poly (d,l-lactic acid)) (PEG-PLA) would allow for efficient drug delivery and could circumvent the low bioavailability associated with oral administration through intravenous injection. The results demonstrated that nanoparticle (NP)–Vanib induced cytotoxicity in cancer cells by inhibiting angiogenesis effectively [95]. While these studies have demonstrated greater efficacy in combination with additional therapies, such an approach warrants additional studies to investigate other therapeutic agents in other cancers as well.

### 3.8. Cytokines

Many antitumor responses that target cancer cells are often suppressed by the interaction of PD-1 and PD-L1, which inhibit T cell activity and the release of various cytokines (i.e., IFN-γ and IL-2). Additionally, proinflammatory cytokine production by CD8+ T cells has been found to be associated with an obesity-induced hepatic type I interferon (INF-1) response in the early stages of nonalcoholic steatohepatitis (NASH) [97], which in turn has been shown to contribute to hepatocyte damage. In addition, higher expression of proinflammatory cytokines has been shown as part of the imbalance of helper T cells and Tregs, which all in turn promote the progression of NAFLD [98,99].

Recently, Zhu et al. reported that using AFP-specific T cell receptor-engineered T cells (AFP-TCR-T) armed with a novel IL-21 receptor appeared to be a promising target to improve adoptive T cell therapy efficacy in HCC. In addition, the receptor IL-21R-TCR-T demonstrated activation-induced proliferation and superior antitumor activity compared to conventional AFP-TCR-T [100]. 

In addition, there are cytokine-induced killer (CIK) cells that are a mix of T cells and NK cells which are formulated from a patient’s peripheral blood mononuclear cells with IL-2 and a CD3 antibody. These cells are more specific for tumor cells and can lead to tumor cell death. In a phase III trial with 230 HCC patients comparing CIK and no adjuvant treatment, those who underwent CIK treatment displayed a median recurrence-free survival of 44 months [101].

## 4. Conclusions

Various completed studies have greatly increased treatment options for HCC patients while some await results (NCT03841201 and NCT0393695); others like the assessment of regorafenib with pembrolizumab (NCT04696055) were discontinued due to the drugs not achieving the primary efficacy endpoints and displaying high frequency of adverse effects. Despite various challenges, immunotherapy advancements have begun focusing on personalizing treatment options for racially distinct cohorts. While there have been increasing advances in the treatment of HCC, additional studies focusing on racially distinct populations must be conducted to address the health disparities in HCC treatment. HCC can escape recognition and detection by various mechanisms which can often hinder current treatment options and impede the patient’s best chance of a cure either through combination therapies, resection, or additional systemic treatments. Despite those advances, it is imperative to continue research into the various evasive mechanisms, the tumor microenvironment, how racially distinct populations respond to immunotherapy, and how their various etiologies influence patient outcomes. This could potentially lead to less recurrence, less mortality, and increased overall survival in HCC patients.

## Figures and Tables

**Table 1:** Ongoing clinical trials for immune checkpoint inhibitors.

Ongoing Clinical Trials of Immune Checkpoint Inhibitors			
NCT Number	Immune Checkpoint Inhibitors	Phase	Recruitment Status
NCT04696055	Pembrolizumab + Regorafenib	Completed	Completed
NCT05178043	Nivolumab + GT90001	Phase II	Active; Not recruiting
NCT05048017	Regorafenib + PDL1 inhibitor	Phase II	Recruiting
NCT04183088	Tislelizumab + Regorafenib	Phase II	Recruiting
NCT05086692	MDNA11 + ICI	Phase I & II	Recruiting
NCT04050462	Nivolumab + Cabiralizumab + BMS-986253	Phase II	Active; Not recruiting
NCT03893695	GT90001 + Nivolumab	Completed	Completed
NCT03682276	Ipilimumab + Nivolumab	Phase I & II	Recruiting
NCT05257590	Nivolumab + CVM-1118	Phase II	Recruiting
NCT04567615	Nivolumab + Relatlimab	Phase II	Active; Not recruiting
NCT03841201	Lenvatinib + Nivolumab	Completed	Completed
NCT01658878	Nivolumab + Ipilimumab + Sorafenib + Cabozantinib	Phase I & II	Active; Not recruiting
NCT04039607	Nivolumab + Ipilimumab + Sorafenib + Lentvatinib	Phase III	Active; Not recruiting
NCT04170556	Regorafenib + Nivolumab	Phase I & II	Active; Not recruiting
NCT03539822	Cabozantinib + Durvalumab + Tremelimumab	Phase I & II	Active; Not recruiting
NCT04102098	Atezolizumab + Bevacizumab	Phase III	Active; Not recruiting
NCT04912765	Neoantigen + Dendritic cell vector + Nivolumab	Phase II	Recruiting
NCT03829436	TPST-1120 + Nivolumab	Phase I & II	Completed
NCT03170960	Cabozantinib + Atezolizumab	Phase I & II	Active; Not recruiting
NCT05176483	XL092 + Nivolumab + Ipilimumab + Relatlimab	Phase I	Recruiting
NCT05337137	Relatlimab + Nivolumab + Bevacizumab	Phase I & II	Recruiting
NCT03439891	Nivolumab + Sorafenib	Phase II	Active; Not recruiting

Abbreviations: ICI; immune checkpoint inhibitor, PD-1; programmed cell death protein 1.

## Data Availability

The data presented in this study are available in this article.
